# Multiple Dipole Source Position and Orientation Estimation Using Non-Invasive EEG-like Signals

**DOI:** 10.3390/s23052855

**Published:** 2023-03-06

**Authors:** Saina Namazifard, Kamesh Subbarao

**Affiliations:** Department of Mechanical and Aerospace Engineering, The University of Texas at Arlington, 500 W. First St., Arlington, TX 76019, USA

**Keywords:** dipole, EEG, head, inverse-problem, localization, model, non-invasive, signal, source

## Abstract

The problem of precisely estimating the position and orientation of multiple dipoles using synthetic EEG signals is considered in this paper. After determining a proper forward model, a nonlinear constrained optimization problem with regularization is solved, and the results are compared with a widely used research code, namely EEGLAB. A thorough sensitivity analysis of the estimation algorithm to the parameters (such as the number of samples and sensors) in the assumed signal measurement model is conducted. To confirm the efficacy of the proposed source identification algorithm on any category of data sets, three different kinds of data-synthetic model data, visually evoked clinical EEG data, and seizure clinical EEG data are used. Furthermore, the algorithm is tested on both the spherical head model and the realistic head model based on the MNI coordinates. The numerical results and comparisons with the EEGLAB show very good agreement, with little pre-processing required for the acquired data.

## 1. Introduction

The human brain composed of neurons that connect with each other via electrical signals. One can record and measure these activities using an electroencephalogram (EEG). An essential use of the EEG is in locating the generating source of these signals, usually approximated by dipoles. This is important because, in some particular circumstances, neurons may not function optimally and could make the equivalent dipole generate abnormal signals. This could be the result of seizures or other brain disorders. In order to isolate such disorders, the challenge is to find a non-invasive way to locate the anomalous source. In [1], the authors addressed the location of abnormality for mildly depressed patients. In this case, only few regions were associated with depression. Thus, to treat these disorders, source localization is crucial and vital in clinical subjects exhibiting such neural activity [2].

EEG signals’ source localization has been extensively studied. Cohen et al. in [3] additionally compared the accuracy of using EEG signals versus magnetoencephalogram (MEG) signals and showed that EEG signals are as useful as MEG signals for source localization problems. Furthermore, in [4], the authors described trend source localization methods using the finite element method for modeling the human head, as well as defined a time-slices approach.

Among all the studies, there are two important steps for source localization: (1) The forward model for EEG signal approximation; and (2) The inverse problem for locating the generating source. In the forward problem, the electrode potentials are calculated based on the given source properties. Many review articles address different forward problem approaches pertaining to source localization as in [5]. Other studies such as [6,7] focused on a specific forward problem approach such as implementing the boundary element method (BEM) and its effect on source localization error. Moreover, in [8], the effect of forward model errors, and the way to remove them using a Monte Carlo approach is also discussed. The inverse problem, on the other hand, is solved when the EEG signals are available and measured by electrodes, and the goal is essentially to estimate the signal properties. There are several inverse problem approaches available to find the source location of the signal generators or the dipoles. Robert Grech and their co-authors present a comprehensive review of the approaches, including minimum norm estimates, low-resolution electromagnetic tomography (LORETA), local auto-regressive average (LAURA), adaptive standardized LORETA/FOCUSS (ALF), and multiple signal classification (MUSIC) [9]. Among these mentioned methods, MUSIC is widely cited (and used) and the main idea is based on the subspace decomposition technique [10]. In other words, this technique tries to select the best signal subspace that works properly for the forward model. Ref. [11] builds upon this by introducing a new algorithm based on QR decomposition, and compares it with other available algorithms such as recursively applied and projected MUSIC (RAP-MUSIC) [12]. One of the recent studies showed promising results using the $L_{2}$ norm to solve the underlying ill-posed inverse problem based on Bernoulli Laplacian Priors [13].

Besides the need for robust mathematical algorithms to solve the source localization problem from EEG signals, there is also the need to model the propagation of the signal through the brain media (matter) before the signal is picked up by the sensors. Among the key factors that affect the signal quality is the conductivity of the brain matter. For example, refs. [14,15] implement non-uniform conductivity for the head model. In similar studies, researchers have mostly considered different conductivity for the skin, compact bone, spongy bone, and the brain. Despite these models’ popularity, other novel methods exist to solve the forward problem more accurately. For instance, ref. [16] presents a two-volume integral equation for the inhomogeneous and anisotropic forward problem, which is more precise than common differential equation-based available methods.

Another important aspect that impacts the source localization solution is the number, and the distribution of the sensors. Many studies are available that show the sensitivity of the solution to different number of sensors, as well as examine the number of sensors needed to have a precise solution [17,18]. There are also studies such as [19] that quantify the mislocation of sensors, considering them as random variables. However, these studies do not address the effect of the distance from electrodes to the signal resource nor the received signal strength (RSS) attenuation in the source localization problem.

In this paper, a new algorithm will be introduced to estimate the dipoles’ properties, namely strength, location, and orientation, using EEG-like signals. The main contribution is the development of a mathematical model that can be utilized in the inverse solution to determine the source location and orientation. The developed model considers randomly distributed conductivity in the ‘head model’ as well as randomly distributed sensor locations. This model captures a wide variety of signals received from head models (Finite Element Method-based on specified conductivities for matter inside). The models are verified using the tools from Brain Electrical Source Analysis (BESA) (https://www.besa.de/products/besa-research/features/head-model-selection/, accessed on 15 January 2023).

In the following sections of this paper, the inverse problem is setup by describing the components of a measurement model. Following this, the solution methodology is presented that describes a constrained optimization approach to solve the inverse problem. The introduced algorithm is applied to three different datasets. First, synthetic data generated by a forward model is utilized to assess the accuracy of the source properties estimation. Second, two different clinical datasets, including a seizure, are considered. Eventually, all the results are compared using a widely available tool-EEGLAB [20].

## 2. EEG Measurement Model

This section describes the EEG measurement model that is utilized to generate synthetic EEG measurements.

### 2.1. Head Model

**Figure 1:**
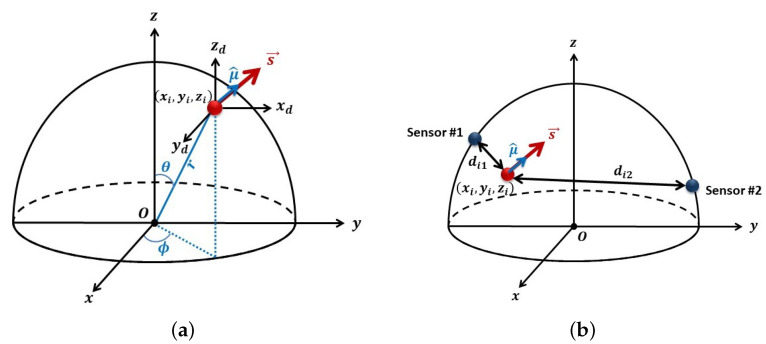
Half-sphere head model: (**a**) spherical coordinates and (**b**) an example of two sensors in the presence of the ith dipole.

**Figure 2:**
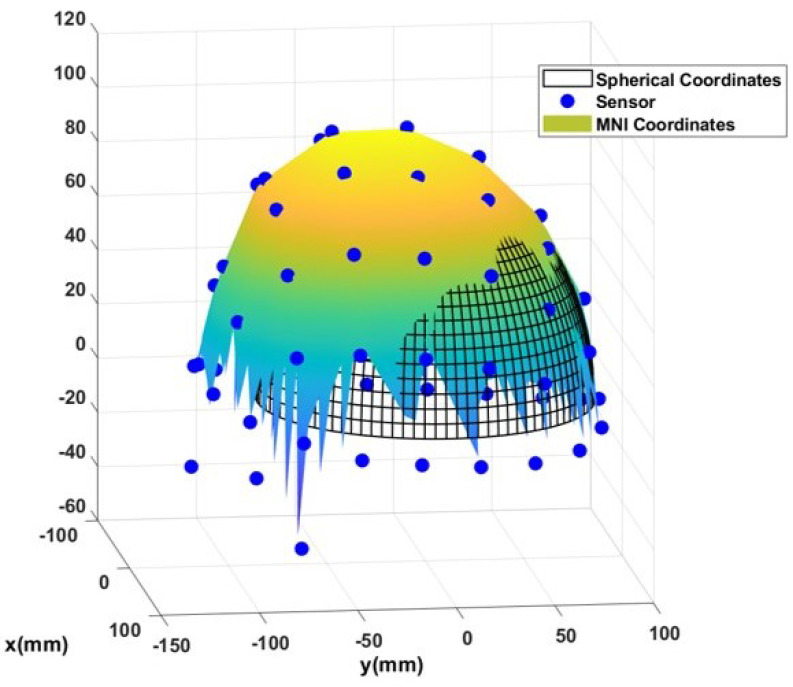
MNI and spherical head model in a same figure.

In this study, two different head models are evaluated: (1) a hemi-spherical (half sphere) head model, as shown in Figure 1; and (2) a realistic head model based on the Montreal Neurological Institute (MNI) coordinates. A comparison of these two head models is presented in Figure 2. Note that in both head models, +y axis passes through the nasion, and the +x axis passes through the right ear. First, let us consider a half sphere as a brain model which is shown in Figure 1.

According to Figure 1a, the location of any ith dipole with respect to the origin by utilizing spherical coordinates can be presented in a vector $l_{i}={\left[l_{x_{i}}\;l_{y_{i}}\;l_{z_{i}}\right]}^{T}$, given as:

(1) $$\left\{\begin{array}{ccc} l_{x_{i}} & = & r_{i}\sin\theta_{i}\cos\phi_{i}\\ l_{y_{i}} & = & r_{i}\sin\theta_{i}\sin\phi_{i}\\ l_{z_{i}} & = & r_{i}\cos\theta_{i} \end{array}\right.$$

In this study, the radius of the brain spherical model is considered as r=10 cm, and thus, $r_{i}\in \left[0,\;10\right)$. Moreover, the two angles are defined as $\theta_{i}\in \left[0,\frac{\pi }{2}\right)$ and $\phi_{i}\in \left[0,2\pi \right)$ to cover the whole half-sphere head model.

Similarly, the jth sensor location is described as $w_{j}={\left[w_{x_{j}}\;w_{y_{j}}\;w_{z_{j}}\right]}^{T}$, obtained using

(2) $$\left\{\begin{array}{ccc} w_{x_{j}} & = & r\sin\theta_{j}\cos\phi_{j}\\ w_{y_{j}} & = & r\sin\theta_{j}\sin\phi_{j}\\ w_{z_{j}} & = & r\cos\theta_{j} \end{array}\right.$$

Figure 1b illustrates the relation of the ith dipole and two selected sensors.

The vector $s_{i}=s_{i}\;\mu_{i}$$\in R^{3\times 1}$ is the dipole signal strength (with units as Coulomb-meter), $\mu_{i}$$\in R^{3\times 1}$ is the unit vector denoting the orientation of the dipole, and $s_{i}$∈R is the magnitude. An alternate representation could be $s_{i}={\left[s_{x_{i}}\;s_{y_{i}}\;s_{z_{i}}\right]}^{T}$. Note, the latter requires three parameters to specify the *i*-th dipole as opposed to four parameters for the former. However, for this study, the 4 parameter representation is used since it provided better estimates of the dipole orientation.

Figure 1b also shows the distance between a dipole and two different sensors, which is denoted by $d_{ji}$ where *j* is the sensor number, and *i* the dipole number.

### 2.2. EEG Signal Measurement

The mathematical model for the measured EEG signal is adapted from [9,21], together with a measurement noise model ($n_{j}$), as shown in Equations (3) and (4) reflects the inverse square field from the Biot–Savart law as in [9]. This model represents the values of measured signals (in μV) that would have been obtained using electrodes located on the patient’s scalp and is shown below:

(3) $$f_{j,i}=g_{j,i}s_{i}\mu_{i}+n_{j}$$

n$\in R^{m\times 1}$ represents the Gaussian white noise process with some specified covariance. Thus, $f_{j,i}\in R$ is the signal strength of the ith dipole received at the jth sensor and $g_{j,i}$ is given as

(4) $$g_{j,i}=\frac{1}{4\pi \zeta }\left[\frac{w_{x_{j}}-l_{x_{i}}}{d_{ji}^{3}}\frac{w_{y_{j}}-l_{y_{i}}}{d_{ji}^{3}}\frac{w_{z_{j}}-l_{z_{i}}}{d_{ji}^{3}}\right]$$

where $d_{ji}$ shows the distance between the ith dipole and the jth sensor.

Furthermore, denote $G_{i}\in R^{m\times 3}$ as the gain matrix for the ith dipole with *m* sensors, and constant brain conductivity ζ (μS/cm) [11]. Thus:

(5) $$G_{i}=\frac{1}{4\pi \zeta }\left[\begin{array}{ccc} \frac{w_{x_{1}}-l_{x_{i}}}{d_{1i}^{3}} & \frac{w_{y_{1}}-l_{y_{i}}}{d_{1i}^{3}} & \frac{w_{z_{1}}-l_{z_{i}}}{d_{1i}^{3}}\\ \vdots & \vdots & \vdots \\ \frac{w_{x_{m}}-l_{x_{i}}}{d_{mi}^{3}} & \frac{w_{y_{m}}-l_{y_{i}}}{d_{mi}^{3}} & \frac{w_{z_{m}}-l_{z_{i}}}{d_{mi}^{3}} \end{array}\right]$$

The EEG signals, as received by the *m* sensors, are then compactly represented as

(6) $$F_{i}=s_{i}G_{i}\mu_{i}+n$$

This model illustrates the relationship between the dipole properties and the collected EEG signals, considering the noise of the sample collecting process. Note that $F\in R^{m\times n}$.

To account for the fact that the conductivity of the brain material is non-uniform, we propose a piece-wise constant conductivity to account for the soft matter, as well as the skeletal tissue before the dipole signal is received at the sensor. This is modeled as follows to present a more realistic conductivity:

(7) $$f_{j,i}=\rho_{j,i}s_{i}\left(\frac{1}{4\pi \zeta_{j,i}}\right)g_{j,i}\mu_{i}+n_{j}$$

In what follows, each of the terms introduced in Equation (7) such as $\zeta_{j,i}$ and $\rho_{j,i}$ will be elaborated upon.

**Figure 3:**
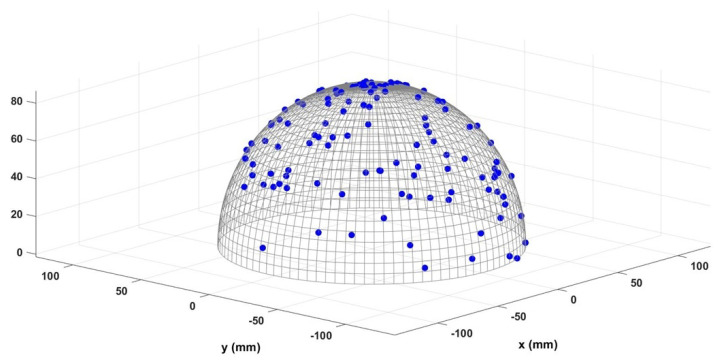
Uniform random distribution of sensors located in the hemisphere head model.

**Figure 4:**
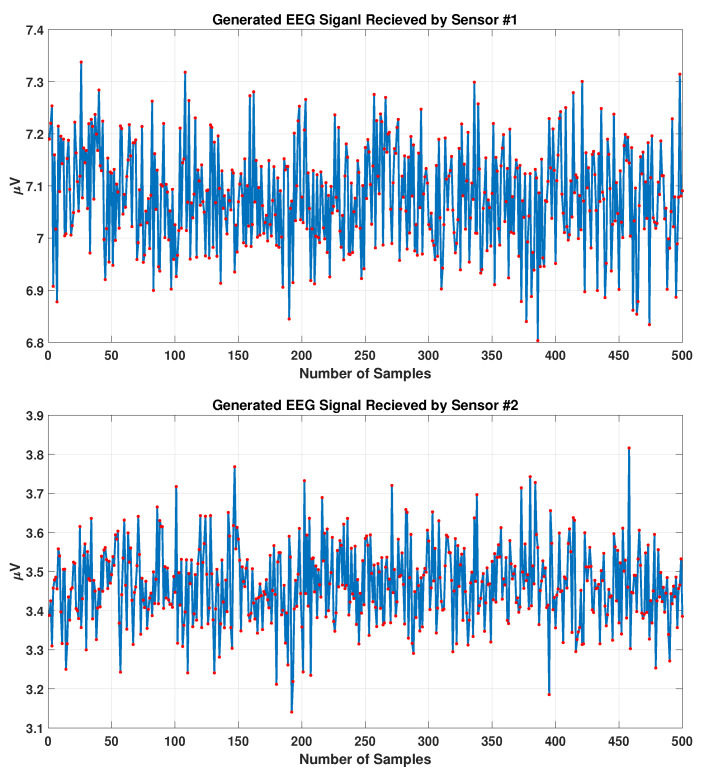
Synthetic EEG signals recorded by two different sensors. Signal 1 has a higher absolute mean strength compared to signal 2.

- Variable conductivity ($\zeta_{j,i}$) within the head model: It is assumed that a dipole can be located anywhere in the brain (half-sphere head model), and the sensors are located on the patient’s scalp. This means the generated signals from dipoles pass through different parts of the head, such as the brain’s soft tissue, the spongy bone, the compact bone part, and the skin, to reach the sensors. Since each of these materials have different conductivities, the signal conduction is affected accordingly. In order to model this changing conductivity, the $\zeta_{j,i}$ is modeled as a uniformly distributed random variable between 0.1 and 0.9 all the way through the head (including the soft parts to the hard parts such as bone).
- Sensor distribution: Several methods are available to locate the electrodes on the patient’s scalp, usually named by numbers indicating the standard locations. For instance, the two most popular sensor distributions are 10–10 and 10–20 [22]. This study aimed to find a precise estimation of the dipole properties, regardless of how the sensors are distributed. In order to fulfill this goal, a random distribution of sensors is considered in this paper. Note that the sensors are uniformly randomly distributed over the scalp. Figure 3 illustrates a sample of uniform random distribution for 128 sensors.
- The adjacency of the dipoles to the sensors and the varying received signal strength (RSS) as a function of the location of the dipoles with respect to the sensors $\left(\rho_{j,i}\right)$: the accuracy of the collected signals is directly related to the distance between the source and the sensor. In this paper, the location of sensors is assumed to be uniformly randomly distributed. Thus, the distance between the dipole to each sensor can be varied from 0 (co-located source and sensor) to the brain’s diameter (i.e., 2r=20 cm in this paper). Thus, greater proximity to the sensor translates into a better RSS value of the EEG signal. In order to consider this, a distance-based signal strength attenuation term $\rho_{j,i}$ is modeled as follows
  (8) $$\rho_{j,i}=-\alpha d_{ji}+\beta$$
  where α and β are constants and chosen such that the coefficient $\rho_{j,i}$ lies within 0.01 and 1.0. Note that the units of $\rho_{j,i}$ are the same as those of the charge, i.e., Coulomb.
  Note that these coefficients eliminate the effect of weak and noisy data and keep the high strength signals. In order to illustrate this concept, consider the following example. For the closest distance (the dipole is right under the sensor), the collected value will be the same as the real value generated by the dipole, which means that the coefficient is equal to exactly 1.0. With the same approach, for the longest distance (the brain diameter), the collected value will be 0.01 times the real value, which means that one can neglect it.
  The synthetic signals generated using the aforementioned EEG measurement model are shown in Figure 4.

**Figure 5:**
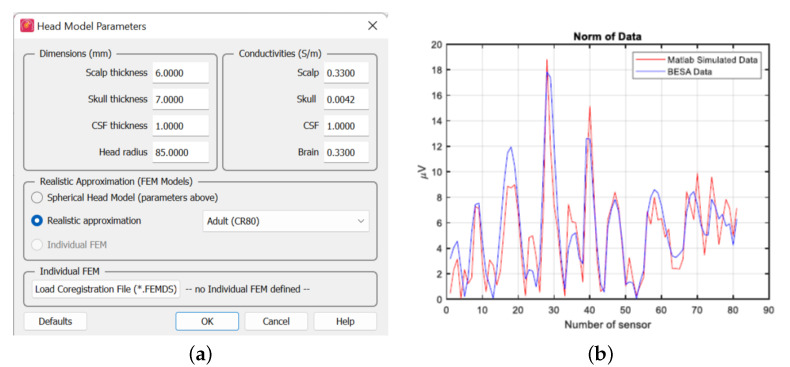
BESA comparison: (**a**) model parameters for the BESA data; (**b**) comparison of BESA data with proposed signal model.

In this paper, to illustrate the effectiveness of the measurement model as well as the estimation algorithm, it is assumed that the dipoles are fixed in orientation and magnitude. The head is modeled as a half-sphere with a diameter of 20 cm as it is suggested in [23]. Figure 5 shows a comparison of BESA (https://www.besa.de/products/besa-research/features/head-model-selection/, accessed on 15 January 2023) data, with specific head model parameters (conductivities) shown in Figure 5a against that synthesized using our method assuming that ζ is uniformly distributed between 0.2 and 0.4 S/m. The results are in very good agreement. Henceforth, all synthetic data were generated using the proposed mathematical model following this verification.

## 3. Problem Statement

Succinctly stated, the problem that is solved in this paper is as follows. “Assuming multiple dipole sources (*n*) with fixed orientations and strengths, and given *m* noisy synthetic EEG signal measurements, we seek to find an accurate estimate of the unknown parameters that characterize dipoles, such as their distinct locations, orientations, and the strengths of the dipoles using a simple phenomenological measurement model provided in Equation (6)”.

## 4. Solution Methodology

The parameters to be estimated for the *i*-th dipole are denoted compactly as the vector $p_{i}\in R^{7\times 1}$:

$$p_{i}={\left[l_{x_{i}}\;l_{y_{i}}\;l_{z_{i}}\;\mu_{x_{i}}\;\mu_{y_{i}}\;\mu_{z_{i}}\;s_{i}\right]}^{T}$$

The above-mentioned unknowns all appear in the right side of Equation (6). Thus, the inverse problem is solved to obtain the unknown components in the p vector.

Among the different approaches to solving an inverse problem, this paper chooses an optimization method where the $L_{2}$ norm of the estimation error is minimized. The estimation error is defined as the difference in the real measured signals and those predicted by the estimated values of the unknown parameters using the measurement model in Equation (6).

$${\hat{p}}_{i}={\left[{\hat{l}}_{x_{i}}\;{\hat{l}}_{y_{i}}\;{\hat{l}}_{z_{i}}\;{\hat{\mu }}_{x_{i}}\;{\hat{\mu }}_{y_{i}}\;{\hat{\mu }}_{z_{i}}\;{\hat{s}}_{i}\right]}^{T}$$

The predicted value of the EEG signal from the parameter estimates is given by

(9) $${\hat{F}}_{i}\left({\hat{p}}_{i}\right)={\hat{s}}_{i}{\hat{G}}_{i}{\hat{\mu }}_{i}$$

The objective of the inverse problem is to minimize the weighted $L_{2}$ norm of the measurement residuals, shown as:

(10) $$J=\frac{1}{2}\sum_{i=1}^{i=n}{\left(F_{i}\left(p_{i}\right)-{\hat{F}}_{i}\left({\hat{p}}_{i}\right)\right)}^{T}W\left(F_{i}\left(p_{i}\right)-{\hat{F}}_{i}\left({\hat{p}}_{i}\right)\right)$$

where W is a symmetric weighting matrix. In this paper, W is chosen to be an identity matrix. The cost function is augmented with the unit norm constraint of the dipole orientation, i.e., $∥{\hat{\mu }}_{i}{∥}_{2}=1$.

$$J_{a}=J+\sum_{i=1}^{i=n}\left(\lambda_{i}\left(∥{\hat{\mu }}_{i}∥-1\right)\right)$$

where $\lambda_{i}$ is the Lagrange Multiplier corresponding to the ith dipole orientation constraint in the equation above. Note that the procedure for solving this problem would be to set up the necessary conditions (from the gradient of the cost), and determine an update for the parameters from one iteration to the next using the gradient and the Hessian (second derivative of the cost function with respect to the decision variables), and the application of the Karush–Kuhn–Tucker (KKT) conditions. These procedures are built into a nonlinear solver such as ‘fmincon’ in the Optimization Toolbox of MATLAB, which allows for an explicit specification of the cost to be minimized, nonlinear constraints that the decision variables satisfy, as well as any bounds on the decision values that need to be respected. The results obtained are discussed in the next section.

## 5. Simulation Results

Before presenting the detailed simulation results, a thorough sensitivity and characteristics analysis of the solution to the inverse problem with regard to the number of sensors to be employed and the number of samples required to reliably provide a converged solution was performed. The signals generated for the analysis have an average signal-to-noise ratio (SNR) of approximately 20.

### 5.1. Model Sensitivity and Characteristics Analysis Using Synthetic Data and the Spherical Head Model

Table 1 provides the results of the simulation in this study. As shown in the table, the source localization process uses an initial guess and iteratively converges to the actual values. As shown in Table 1, the estimated value is very accurate for all three selected dipoles, indicating that the source localization algorithm is consistent.

**Figure 6:**
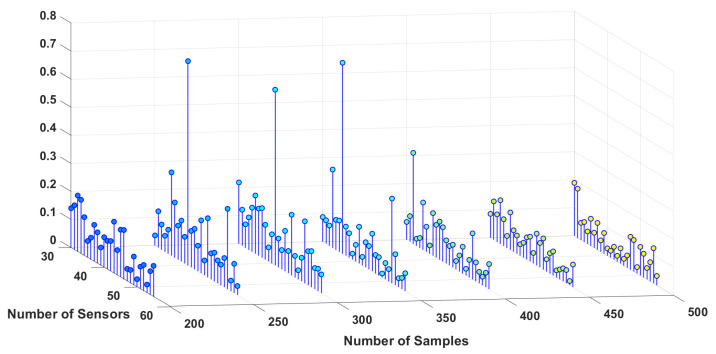
Error percentage of estimated variables in terms of number of sensors and samples, Dipole number 1.

**Figure 7:**
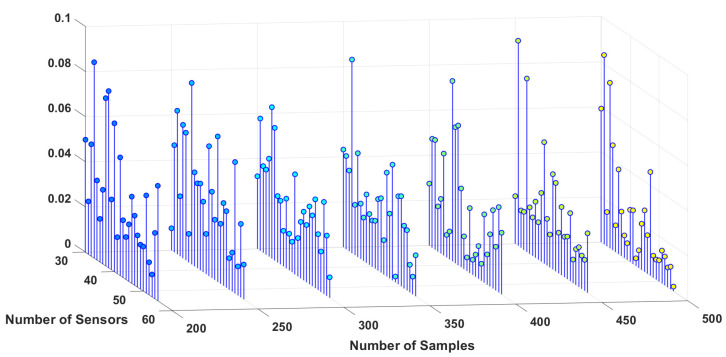
Error percentage of estimated variables in terms of number of sensors and samples, Dipole number 2.

**Figure 8:**
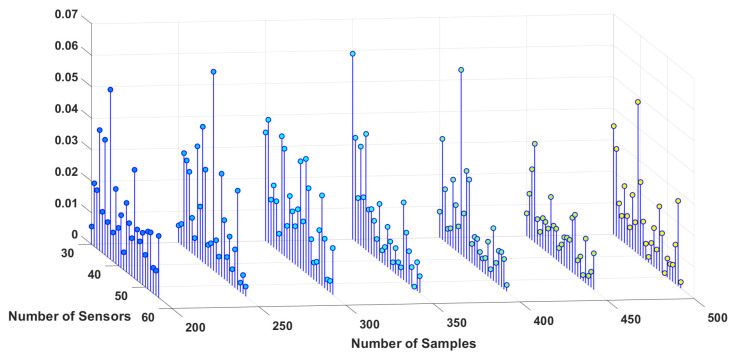
Error percentage of estimated variables in terms of number of sensors and samples, Dipole number 3.

To study the sensitivity of the estimation algorithm to the number of sensors and samples, the simulation is performed for different numbers of sensors, as well as samples for three different dipoles, and the results are shown in Figure 6, Figure 7 and Figure 8. These 3D plots show the changes in the total error percentage of the estimated values in terms of the increasing number of sensors and samples simultaneously. As expected, by growing the number of samples and sensors, the amount of collected data increases. As a result, the estimation of unknown values is more accurate. Moreover, one can determine the least value of error and the corresponding number of samples and sensors. For instance, *in this simulation, 48 sensors and 350 samples gave the least error percentage*.

In order to study the effect of the distance between dipoles and sensors, three different locations are chosen for the simulation, one deeper in the brain, one closer to the scalp and sensors, and one dipole is chosen somewhere in between the locations of the other two. Based on the estimation algorithm described in the previous section, sensors receive weaker signals from the deeper dipoles. This leads to a higher amount of estimation error for the deeper dipoles, i.e., dipole #1 in this study. Considering this information regarding the location of the dipoles, one can interpret Figure 6, Figure 7 and Figure 8 more accurately. This case can be observed by comparing the magnitude of the estimation error for each dipole from these three figures.

**Figure 9:**
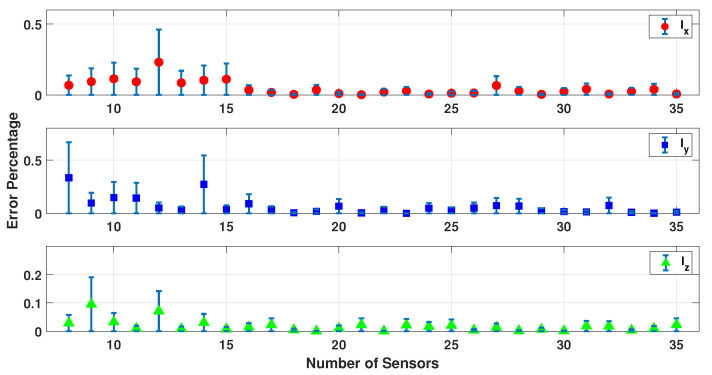
Error percentage of a dipole’s location for different sensors and 500 samples.

**Figure 10:**
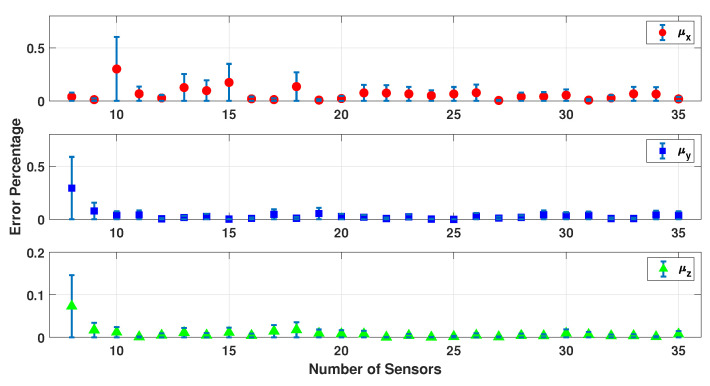
Error percentage of a dipole’s orientation for different sensors and 500 samples.

**Figure 11:**
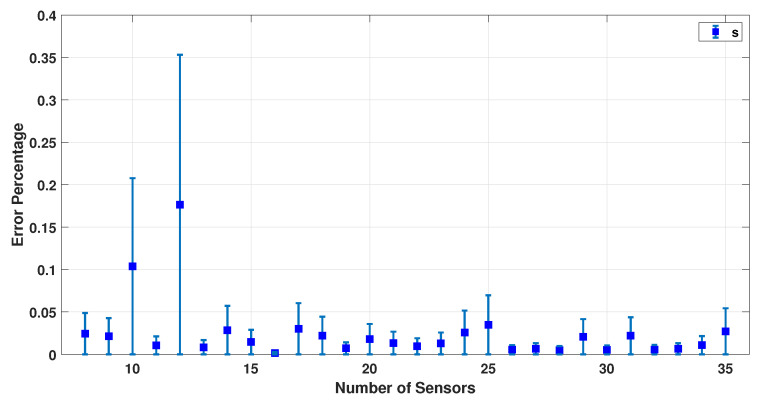
Error percentage of a dipole’s magnitude for different sensors and 500 samples.

**Figure 12:**
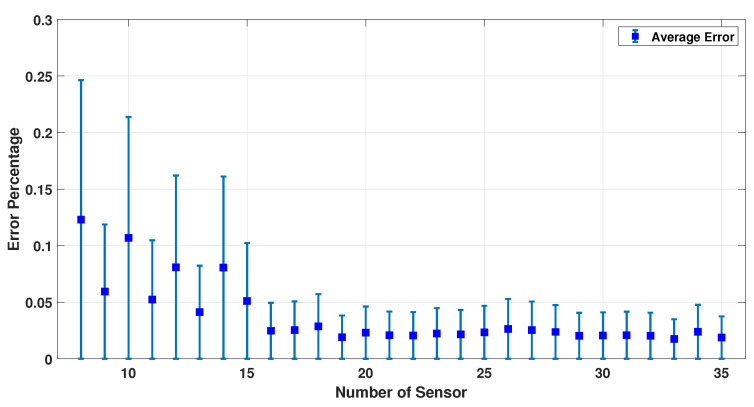
Average error percentage for different sensors and 500 samples.

**Figure 13:**
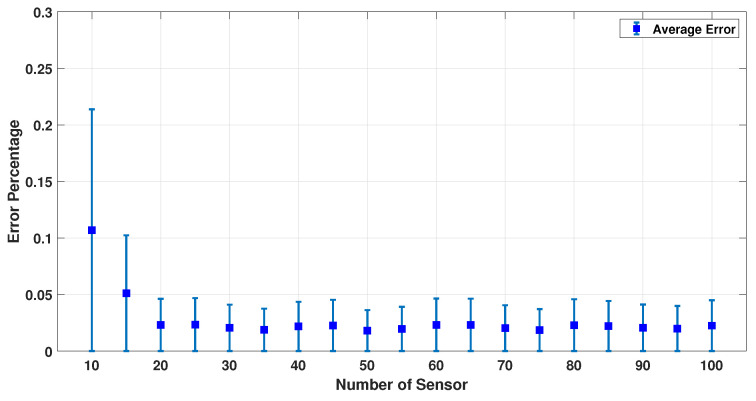
Average error percentage for different sensors (10–100) and 500 samples.

Figure 9, Figure 10, Figure 11, Figure 12 and Figure 13 present the error percentage for estimating each variable in the parameter vector $p_{i}$ separately, where the error bar is the confidence interval value. It was mentioned previously that the electrodes are randomly located on the patient’s scalp to make sure this simulation is working correctly regardless of the sensors’ location. However, in some cases, the sensors could be located somewhere far from the dipole, and as a result, the collected data are noisier and weaker. Clearly, this happens when the number of sensors is excessively low and they cannot cover the scalp adequately. As a result, the confidence intervals for the low number of sensors are more significant. In other words, if the small number of sensors are located in some place near the dipoles, the result is still acceptable.

Figure 9, Figure 10 and Figure 11 show the error percentage for each variable. There is a lower error for each variable in terms of the number of sensors. Thus, it is not possible to choose the optimum number of sensors only using the error in each variable. To address this problem, an average error of all the parameters is studied, as shown in Figure 12 which provides a more robust estimate for the optimum number of sensors required. We note that (a) the minimum error percentage occurs when around 19 sensors are used; and (b) the error percentage reduces as the number of sensors increase (in general). This occurs because a higher number of sensors cover more of the scalp area, and as a result, the collected EEG data have more information. To summarize, Figure 12 represents a converged and consistent value of error after a specific number of sensors (20 sensors) are used. This means a specific amount of collected data are enough to make the model equations well defined, and the estimation problem well posed to find a consistent estimate. Thus, one can use the minimum number of sensors, as indicated by these numerical experiments, and conduct a simpler actual experiment (in terms of cost and computational burden). In order to further test this hypothesis, this simulation was performed using up to 100 sensors, and the result is provided in Figure 13.

**Figure 14:**
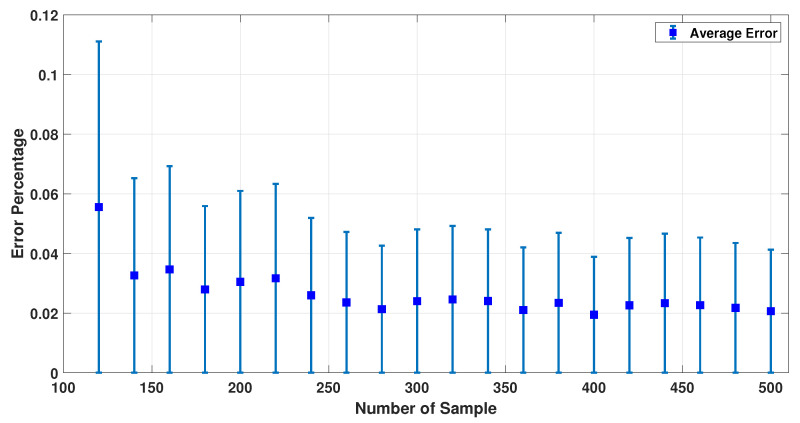
Error percentage for different numbers of samples and 19 sensors.

It was previously discussed that 19 sensors would be good enough for this study to estimate the dipole properties. Similarly, a sensitivity study with respect to the number of samples was carried out. Figure 14 shows the changes in error percentage by increasing the number of samples. It was determined that 400 samples were sufficient to estimate all the dipoles’ parameters.

**Figure 15:**
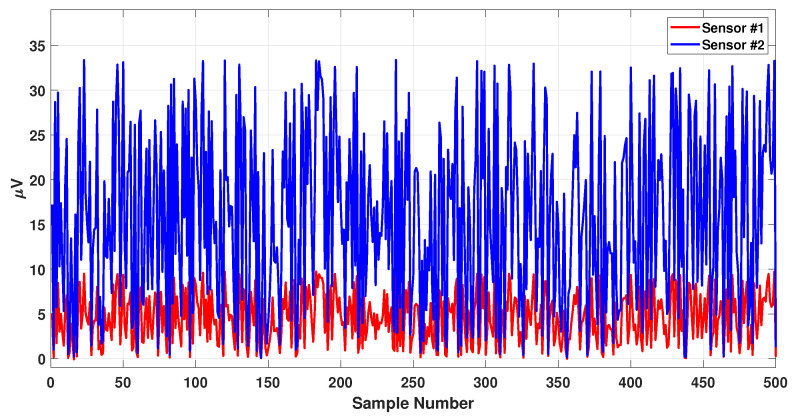
Generating EEG signals recorded by two different sensors.

The effect of distance between the sensor and a dipole is one of the important considerations in this study. In order to see this, two different sensors are considered, one closer to a specific dipole, and the other one far from the same dipole, as was previously shown in Figure 1. The collected EEG signal from these two dipoles is shown in Figure 15. As can be seen from this figure, the sensor collected a stronger EEG signal from the closer dipole (shown in blue) rather than the dipole far from the sensor (shown in red).

In conclusion, the proposed mathematical model for the dipole is very general and captures the key features of signal propagation from the source to the sensor. This simple model allows significant flexibility in terms of medium dependent conduction, signal strength variation due to distance, as well as other unmodeled noises.

### 5.2. Model Performance for Source Localization Using EEGLAB

In this study, the half-sphere head model is used to simplify the simulation. Furthermore, different parts of the brain’s conductivity are assumed to be random numbers between 0.1 and 0.9 as mentioned before in Section 2.

In order to check how precise these assumptions are, one can compare the derived results provided by the present algorithm with the generated results from available open source software such as BESA, Cartool, EEGLAB, etc. [24]. In this study, the EEGLAB [25] software is chosen, with several options to choose the head model. The MNI head model is one of the most accurate ones selected in this study to compare with the spherical head model.

The first step in using EEGLAB is to import the same generated signal used in the previous section. The location of sensors in the MATLAB-generated EEG signal are the standard 64 locations of BioSemi (known as 10–20 standard system), and the same locations will be considered in EEGLAB. The MNI head model in EEGLAB has a head radius of 85 mm. Note that, for having a more accurate comparison, the head model in the previous sections that considered a half sphere is also modified to an 85 mm radius.

**Figure 16:**
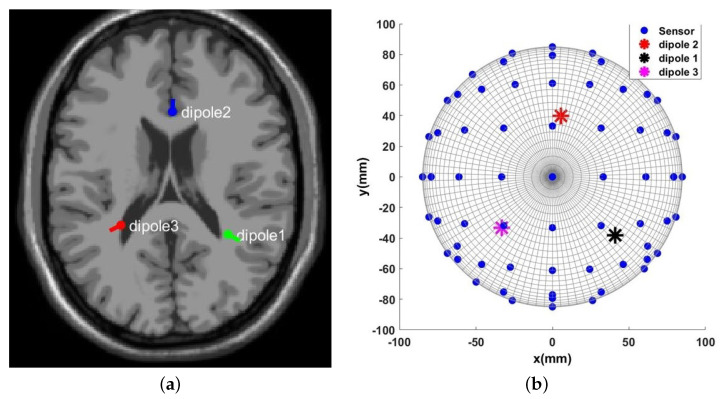
(**a**) Multiple dipoles’ source localization result from EEGLAB where the Talairach coordinates are: dipole 1 (32, −40, 72); dipole 2 (1, 26, 65); and dipole 3 (−27, −35, 73). (**b**) Multiple dipoles’ source localization result from the introduced algorithm.

Note that prior to the source localization step using clinical data in EEGLAB, the user has to reject certain data by visual inspection. *The proposed algorithm (discussed previously) on the other hand does not require this manual step*. Furthermore, since only dipole activities generate the available EEG data, there is no need to denoise or cancel any other ‘artifact’ in this case. The DIPFIT toolbox is used in EEGLAB to generate the source localization results. This MNE-based toolbox is designed by Robert Oostenveld and is available at Fieldtrip [26]. Applying the DIPFIT function in EEGLAB gives the dipole’s location in Talairach coordinates, as shown in Figure 16a. Furthermore, Figure 16a,b are located next to each other to show the similarity of the source localization from the proposed algorithm and the result obtained from EEGLAB. Given the differences between these two approaches and those associated with the representations (Talairach coordinates vs. the Cartesian coordinates), the results are in close agreement. Besides the visual presentation of the results, Table 2 provides the numerical results for the three dipoles located in the head model. The error mentioned in this table is the distance between the actual source and the source localization result in mm, which is defined in Equation (11).

(11) $${\mathrm{error}}_{i}=\sqrt{{\left(l_{x_{i}}-{\hat{l}}_{x_{i}}\right)}^{2}+{\left(l_{y_{i}}-{\hat{l}}_{y_{i}}\right)}^{2}+{\left(l_{z_{i}}-{\hat{l}}_{z_{i}}\right)}^{2}}$$

Note that for all three dipoles, the error is significantly lower for the present algorithm compared to the EEGLAB result. We thus verify that the constrained nonlinear least-squares-based source localization method’s performance agrees well with that produced by EEGLAB.

### 5.3. Characteristics Analysis Using the Clinical Data and the MNI Coordinates

**Figure 17:**
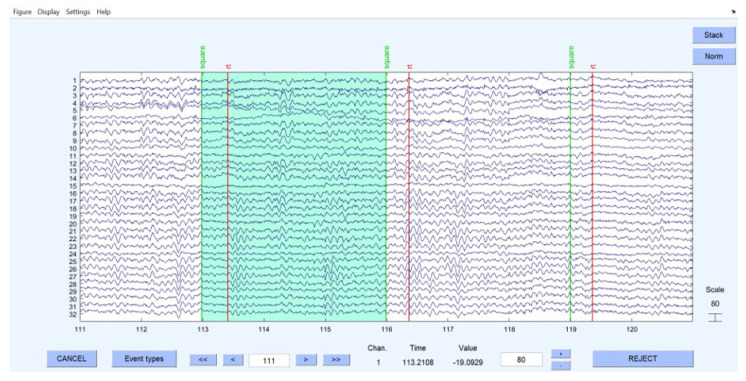
Selected time range from the original dataset.

Now that the efficacy of the proposed algorithm has been proven in the previous sections, one can assess this algorithm using clinical sample data. Therefore, a suggested dataset from EEGLAB is used in this section [20,27]. This dataset belongs to a visual attention task. In this task, each event is a three-second time interval where the subject should press a button right after seeing a square on a screen in front of them. Given the quality of the dataset and the presence of bad quality signals, it is suggested to select good quality time intervals rather than using the whole dataset. Thus, in this study, two different time intervals are selected to quantify the source localization algorithm. As illustrated in Figure 17, one of the selected events is from 113 to 116 s, and the other is from 146 to 149 s.

The selected events are utilized to generate the source localization results on both the EEGLAB software and the introduced algorithm. The EEGLAB source localization algorithm suggests the number of dipole locations to be as many as the channel numbers. In other words, for this specific dataset that was recorded by 30 EEG signals, EEGLAB found 30 possible dipoles that generate this signal. However, only the results with residual variances (RVs) less than 15% are acceptable according to EEGLAB [28]. The residual variance in EEGLAB software is defined based on matching the dipole projection to the EEG electrodes. Based on this definition, it is also important to mention that smaller numbers of RV indicate the most accuracy, and the results are more reliable.

**Figure 18:**
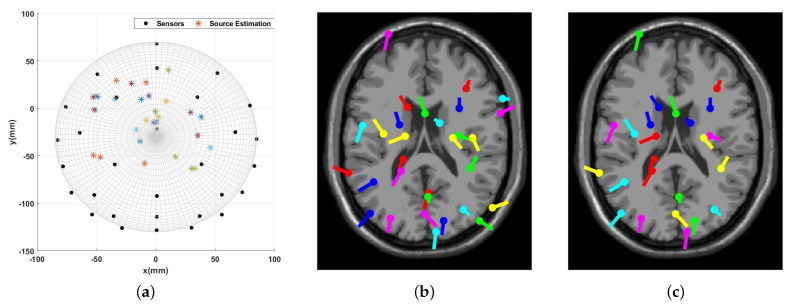
Visual Attention Task (113 s - 116 s): The source localization result from the introduced algorithm vs. EEGLAB. (**a**) MATLAB simulation result; (**b**) EEGLAB result, all estimations; and (**c**) EEGLAB Result, RV >15%.

**Figure 19:**
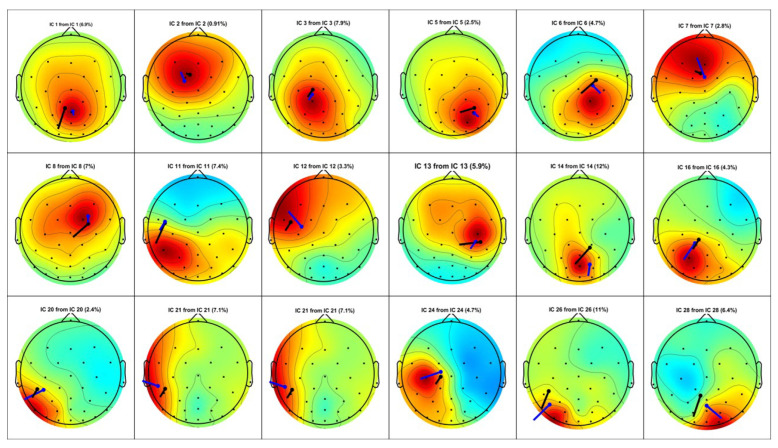
Visual Attention Task (113 s–116 s): Multiple dipoles’ source localization result from the introduced algorithm.

Figure 18 and Figure 19 represent the source localization results for the experiment that started at 113 s and finished at 116 s. Figure 18 illustrates all the dipole estimations simultaneously. Given this general presentation of results, one can see the similarity between the EEGLAB result and the introduced algorithm result. Note that, as mentioned earlier, Figure 18b is the general result by EEGLAB where all the dipole estimations are not necessarily correct. Figure 18c is the final result where any estimation with the RV over 15% is filtered out.

EEGLAB usually utilizes an MRI scan to show the source localization result, making the comparison less convenient. This suggests providing another method to compare the results, and Figure 19 is provided to compare the dipole estimations individually in the same brain map using the Talairach coordinates.

Among the 24 dipoles from EEGLAB results, there are 18 similar results available from the present algorithm. The average distance of the EELAB estimation to the proposed algorithm’s result for these 18 dipoles is only 17.7 mm, and the standard deviation is 7.4 mm. This result clearly illustrates the accuracy of the introduced algorithm when using the clinical data.

**Figure 20:**
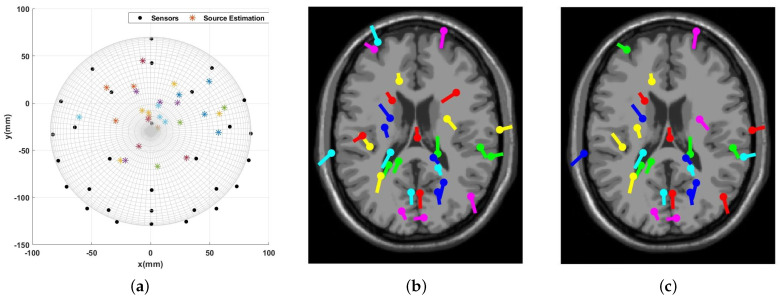
Visual Attention Task (146 s–149 s): The source localization result from the introduced algorithm vs. EEGLAB: (**a**) Proposed algorithm’s result; (**b**) EEGLAB result, all estimations; (**c**) EEGLAB result, RV > 15%.

**Figure 21:**
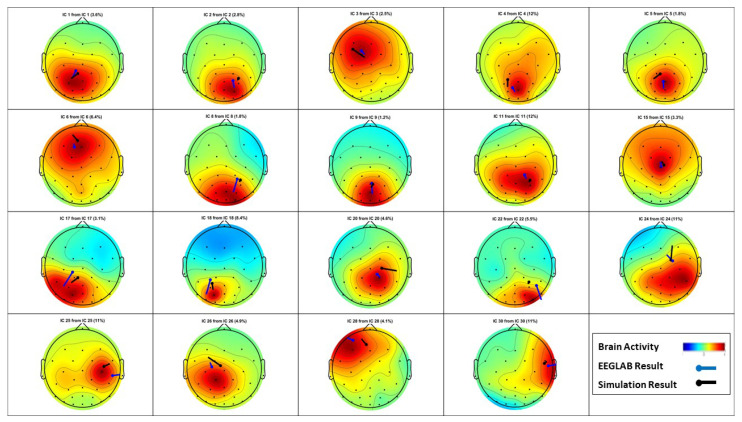
Visual Attention Task (146 s–149 s): Multiple dipoles’ source localization result from the introduced algorithm.

Similarly to the sample data for the 113–116 s time interval, Figure 20 and Figure 21 illustrate the source localization result for the 146–149 s time interval. It should be noted that for this time interval, 27 out of 30 dipole estimations from EEGLAB are acceptable with an RV greater than 15%. Furthermore, the number of similar results from the present algorithm is 19 dipoles.

**Figure 22:**
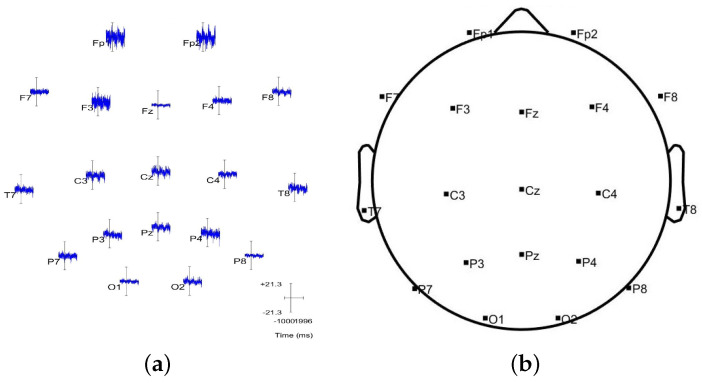
The ERP data are shown for each channel which helps to validate the source localization result. (**a**) Channel data; (**b**) Channel location.

Finally, the algorithm is also tested on clinical data collected from a patient with an active seizure case. The Temple University Hospital provided the EEG data used in this section as their open source database [29]. These data were collected by 19 EEG sensors with the international 10–20 EEG electrodes, as shown in Figure 22b.

**Figure 23:**
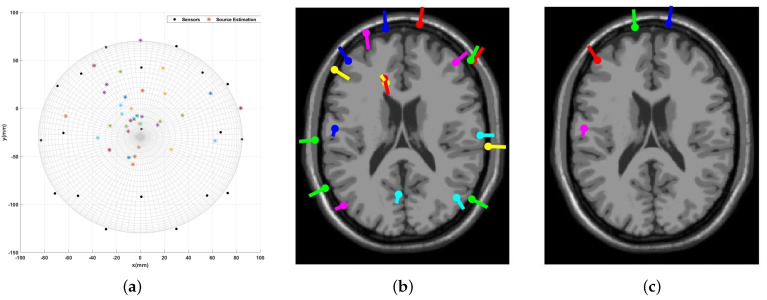
Active Seizure Case: The source localization result from the introduced algorithm vs. EEGLAB. (**a**) Proposed algorithm’s result; (**b**) EEGLAB result, all estimations; (**c**) EEGLAB result, RV > 15%.

**Figure 24:**
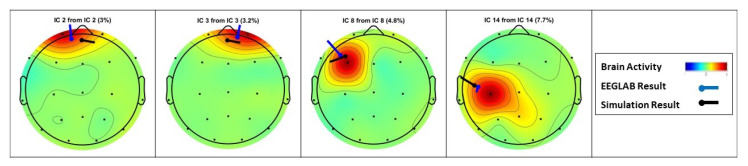
Active Seizure Case: Multiple dipoles’ source localization results from the introduced algorithm.

After applying the EEG source localization to the seizure EEG data, the comparison in Figure 23 shows that the EEGLAB provides 19 estimated dipoles, and only four have an RV > 15%. However, the proposed algorithm results in 35 dipole estimations, including the four dipole results from EEGLAB. Figure 24 presents the four similar results compared individually.

Given a more significant number of dipole estimations from the MATLAB simulation compared to the EEGLAB, the accuracy of this result is illustrated by providing the channel data. As shown in Figure 22, the highest brain activity occurs around sensors Fp1, Fp2, and F3, which matches both the EEGLAB and MATLAB simulation result. On the other hand, there are other channels with noticeable brain activity, such as Cz, C3, Pz, and P4. This means there could be other sources of brain activity around the central area, even though they are not as strong as the frontal head area. These sources are also discerned by the proposed algorithm’s result, as shown in Figure 23a. In other words, the introduced algorithm is more sensitive to all the brain signals and covers a broader range of source localization.

## 6. Conclusions

This study introduces a constrained nonlinear least-squares algorithm to estimate the dipole properties based on the collected EEG signals. The mathematical model for a signal from the dipole is shown to be very flexible and includes features that model piece-wise conductivity, varying received signal strength (RSS), and random sensor distribution on the scalp. This model flexibility enables one to solve the inverse problem for many different head types and/or numbers or sensors and samples. To give an illustration of this fact, this algorithm is applied to two different types of EEG data: (1) synthetic data generated by the forward model; and (2) clinical data. The results emphasize the accuracy and performance of the present algorithm for both EEG signal categories. The source localization error for the synthetic data is less than 0.1 percent. In the case of testing the clinical data, this algorithm works as accurately as EEGLAB, which is illustrated in the figures. The results also show that the presented algorithm performs extremely well for the localization of multiple dipoles and is verified with the results obtained from EEGLAB. Notably, the introduced algorithm can be more sensitive to different sources in the head model. As a result, for some cases, such as the seizure data utilized in this study, the proposed source localization algorithm can find more active dipoles compared to EEGLAB. Future work will focus on further optimizing the number of sensors, decreasing the computation load of source localization algorithms where the FEM and BEM are used, and prototyping the algorithm on a chip that can be embedded in an EEG helmet.

## Figures and Tables

**Table 1:** Source localization result for the multi-dipole case wherein three dipoles are active simultaneously. The simulation is for 250 samples and 36 sensors.

Dipole Param.	Dipole 1			Dipole 2			Dipole 3		
	Real Value	Initial Guess	Est. Result	Real Value	Initial Guess	Est. Result	Real Value	Initial Guess	Est. Result
$l_{x}\;\left(\mathrm{cm}\right)$	2.25	3	2.25	2.37	1	2.37	2.18	1	2.18
$l_{y}\;\left(\mathrm{cm}\right)$	0.82	2	0.82	1.37	−3	1.37	2.18	0	2.18
** $l_{z}\left(\mathrm{cm}\right)$ **	6.58	3	6.58	7.52	3	7.52	8.46	4	8.46
** $\mu_{x}$ **	0.32	0	0.32	0.29	0.43	0.29	0.24	0.43	0.24
** $\mu_{y}$ **	0.12	0	0.12	0.17	−0.75	0.17	0.24	0.25	0.24
** $\mu_{z}$ **	0.29	1	0.94	0.94	0.50	0.94	0.94	0.87	0.94
s(A.cm)	0.1	0.3	0.10	0.2	0.3	0.20	0.3	0.1	0.3

**Table 2:** Numerical results.

Dipole 1					
**Talairach Coord.**	**Real Value**	**Simulation Result**	**Simulation** **Error (mm)**	**EEGLAB Result**	**EEGLAB** **Error (mm)**
X (mm)	9.11	9.13		8	
Y (mm)	32.07	32.06		53	
Z (mm)	38.54	38.52	0.03	46	22.24
**Dipole 2**					
X (mm)	39.96	40.01		59	
Y (mm)	−54.43	−54.39		−53	
Z (mm)	34.37	34.27	0.08	40	19.92
**Dipole 3**					
X (mm)	−15.01	−15.01		−34	
Y (mm)	−29.78	−29.78		−17	
Z (mm)	59.42	59.42	0	69	24.81

## Data Availability

No new data were created or analyzed in this study. Data sharing is not applicable to this article.

## References

[1] Li X., Hu B., Xu T., Shen J., Ratcliffe M. (2015). A study on EEG-based brain electrical source of mild depressed subjects. Comput. Methods Programs Biomed..

[2] Cooper R., Osselton J.W., Shaw J.C. (2014). EEG Technology.

[3] Cohen D., Cuffin B.N., Yunokuchi K., Maniewski R., Purcell C., Cosgrove G.R., Ives J., Kennedy J.G., Schomer D.L. (1990). MEG versus EEG localization test using implanted sources in the human brain. Ann. Neurol. Off. J. Am. Neurol. Assoc. Child Neurol. Soc..

[4] Koles Z.J. (1998). Trends in EEG source localization. Electroencephalogr. Clin. Neurophysiol..

[5] Hallez H., Vanrumste B., Grech R., Muscat J., De Clercq W., Vergult A., D’Asseler Y., Camilleri K.P., Fabri S.G., Van Huffel S. (2007). Review on solving the forward problem in EEG source analysis. J. Neuroeng. Rehabil..

[6] Acar Z.A., Makeig S. (2013). Effects of forward model errors on EEG source localization. Brain Topogr..

[7] Tissari S., Rahola J. (2003). Error analysis of a Galerkin method to solve the forward problem in MEG using the boundary element method. Comput. Methods Programs Biomed..

[8] Liu A.K., Dale A.M., Belliveau J.W. (2002). Monte Carlo simulation studies of EEG and MEG localization accuracy. Hum. Brain Mapp..

[9] Grech R., Cassar T., Muscat J., Camilleri K.P., Fabri S.G., Zervakis M., Xanthopoulos P., Sakkalis V., Vanrumste B. (2008). Review on solving the inverse problem in EEG source analysis. J. Neuroeng. Rehabil..

[10] Schmidt R. (1986). Multiple emitter location and signal parameter estimation. IEEE Trans. Antennas Propag..

[11] Wang Y. (2007). Successive Estimation Method of Locating Dipoles Based on QR Decomposition Using EEG Arrays.

[12] Mosher J.C., Leahy R.M. (1999). Source localization using recursively applied and projected (RAP) MUSIC. IEEE Trans. Signal Process..

[13] Costa F., Batatia H., Chaari L., Tourneret J.Y. (2015). Sparse EEG source localization using Bernoulli Laplacian priors. IEEE Trans. Biomed. Eng..

[14] Montes-Restrepo V., van Mierlo P., Strobbe G., Staelens S., Vandenberghe S., Hallez H. (2014). Influence of skull modeling approaches on EEG source localization. Brain Topogr..

[15] Dannhauer M., Lanfer B., Wolters C.H., Knösche T.R. (2011). Modeling of the human skull in EEG source analysis. Hum. Brain Mapp..

[16] Rahmouni L., Mitharwal R., Andriulli F.P. (2017). Two volume integral equations for the inhomogeneous and anisotropic forward problem in electroencephalography. J. Comput. Phys..

[17] Lantz G., De Peralta R.G., Spinelli L., Seeck M., Michel C. (2003). Epileptic source localization with high density EEG: How many electrodes are needed?. Clin. Neurophysiol..

[18] Song J., Davey C., Poulsen C., Luu P., Turovets S., Anderson E., Li K., Tucker D. (2015). EEG source localization: Sensor density and head surface coverage. J. Neurosci. Methods.

[19] Beltrachini L., von Ellenrieder N., Muravchik C.H. (2011). General bounds for electrode mislocation on the EEG inverse problem. Comput. Methods Programs Biomed..

[20] EEGLAB Tutorial Data. https://eeglab.org/tutorials/tutorial_data.html.

[21] Baillet S., Mosher J.C., Leahy R.M. (2001). Electromagnetic brain mapping. IEEE Signal Process. Mag..

[22] Gordleeva S.Y., Lobov S.A., Grigorev N.A., Savosenkov A.O., Shamshin M.O., Lukoyanov M.V., Khoruzhko M.A., Kazantsev V.B. (2020). Real-Time EEG–EMG Human–Machine Interface-Based Control System for a Lower-Limb Exoskeleton. IEEE Access.

[23] Mosher J.C., Lewis P.S., Leahy R.M. (1992). Multiple dipole modeling and localization from spatio-temporal MEG data. IEEE Trans. Biomed. Eng..

[24] Michel C.M., Brunet D. (2019). EEG source imaging: A practical review of the analysis steps. Front. Neurol..

[25] Delorme A., Makeig S. (2004). EEGLAB: An open source toolbox for analysis of single-trial EEG dynamics including independent component analysis. J. Neurosci. Methods.

[26] Fieldtrip Toolbox. http://www.fieldtriptoolbox.org/.

[27] Makeig S., Westerfield M., Jung T.P., Enghoff S., Townsend J., Courchesne E., Sejnowski T.J. (2002). Dynamic brain sources of visual evoked responses. Science.

[28] Artoni F., Menicucci D., Delorme A., Makeig S., Micera S. (2014). RELICA: A method for estimating the reliability of independent components. Neuroimage.

[29] Obeid I., Picone J. (2016). The temple university hospital EEG data corpus. Front. Neurosci..

